# Cancer stem cells and tumorigenesis

**DOI:** 10.1007/s41048-018-0062-2

**Published:** 2018-08-29

**Authors:** Pingping Zhu, Zusen Fan

**Affiliations:** 1 CAS Key Laboratory of Infection and Immunity, CAS Center for Excellence in Biomacromolecules, Institute of Biophysics, Chinese Academy of Sciences, Beijing, 100101 China; 2 University of Chinese Academy of Sciences, Beijing, 100049 China

**Keywords:** Cancer stem cells, Self-renewal, Signaling pathway, Tumorigenesis, Intervention

## Abstract

Cancer is one of the most serious diseases all over the world, and the cancer stem cell (CSC) model accounts for tumor initiation, metastasis, drug resistance, and relapse. The CSCs within tumor bulk have the capacity to self-renew, differentiate, and give rise to a new tumor. The self-renewal of CSCs is precisely regulated by various modulators, including Wnt/β-catenin signaling, Notch signaling, Hedgehog signaling, transcription factors, chromatin remodeling complexes, and non-coding RNAs. CSCs reside in their niches that are also involved in the self-renewal maintenance of CSCs and protection of CSCs from chemotherapy, radiotherapy, and even endogenous damages. Moreover, CSCs can also remodel their niches to initiate tumorigenesis. The mutual interactions between CSCs and their niches play a critical role in the regulation of CSC self-renewal and tumorigenesis as well. Many surface markers of CSCs have been identified, and these markers become first choices for CSC targeting. Due to heterogeneity and plasticity, targeting CSCs is still a big challenge for tumor elimination. In this review, we summarize recent progresses on the biological features of CSCs and targeting strategies against CSCs.

## History of the cancer stem cell model

Cancer is a leading killer of human health, and more than 10 million patients die of cancer every year (McGuire 2016). Although many tumorous hypotheses and intervention strategies have been revealed, however, the real mechanism of tumor initiation is still elusive. The cancer stem cell (CSC) model fits well with tumor initiation, metastasis, drug resistance, and relapse, which is supported by more and more experimental and clinical data. At present, the CSC model has been accepted by many researchers and clinicians, and will become a promising strategy for tumor intervention in the near future (Shabbir *et al.* 2018; Takebe *et al.* 2011, 2015).

Actually, the concept of CSC model was raised a long time ago. Based on the similarity between cancer and the embryo, Lobstein and Recamier raised an embryonic origin of tumor model in 1829. They thought that tumor originated from proliferating embryonic cells that persisted in adulthood (Krebs 1947). Although this hypothesis fits well with many clinical observations, this hypothesis has not been proved because of technical limitations.

The CSC model was proved for the first time in 1991 in leukemia patients. There are several kinds of leukemia cells, including CD34^+^CD38^−^ cells and CD34^−^CD38^+^ cells. CD34^+^CD38^−^ cells can initiate leukemia efficiently, but CD34^−^CD38^+^ cells cannot, showing the existence of leukemia stem cells (Terstappen *et al.* 1991). Later on, using various tumor models, scientists then found that all the cells in tumor bulk cannot propagate efficiently and only a small subset of tumor cells can initiate new tumors (Shigdar *et al.* 2012). A bunch of surface markers of cancer stem cells have been identified, and CSCs have been identified in many solid tumors up to date (Gopalan *et al.* 2018).

Tumors originate from the CSCs, but what is the origin of CSCs? Several hypotheses have been raised: (1) CSCs are transformed form of differentiated somatic cells; (2) Mutations are accumulated in normal tissue progenitor cells to form CSCs; (3) CSCs originate from dedifferentiation of normal tumor cells. Lineage tracing and single-cell sequencing are good tools to investigate the origin of CSCs. Lineage tracing data by several groups showed that colorectal CSCs are derived from Lgr5^+^ intestinal stem cells (Barker *et al.* 2009; Melo *et al.* 2017; Schepers *et al.* 2012; Shimokawa *et al.* 2017). Taking advantage of single-cell sequencing of bladder CSCs, non-stem tumor cells, and normal bladder cells, Fan lab concluded the multiple sources of bladder CSCs (Yang *et al.* 2017). Single-cell analysis also revealed the heterogeneity of liver CSCs (Zheng *et al.* 2018).

The tumorigenesis models contain the hierarchical models and the stochastic model. Tumors originate from certain cells according to the hierarchical model; whereas the stochastic model assumes that any cells may initiate tumors as a result of mutation or other oncogenic factors (Quail *et al.* 2012). Both models were proved by massive experimental and clinical data, which largely confuse the understanding of tumorigenesis. The standard CSC model belongs to the hierarchical model. According to the classical CSC model, tumors are formed as the result of CSC differentiation, and non-CSCs die of clonal exhaustion (Greaves 2013). However, dedifferentiation occurs in some differentiated epithelial cells to form CSCs, and the dedifferentiation can be hierarchical or stochastic (Chaffer and Weinberg 2015). In liver CSCs, Yap1 activation is prerequisite for self-renewal and cell-fate determination. Yap1 deficiency in liver CSCs can convert them into non-CSCs, and differentiated liver cancer cells became liver CSCs when Yap1 is enforcedly overexpressed (Zhu *et al.* 2016b). The cell-fate switch between CSCs and non-CSCs confirms the plasticity of CSCs, which may combine the hierarchical and stochastic tumorigenesis models together.

## Biological characteristics and study strategies of CSCs

The CSCs within tumor bulk display the capacity to self-renew, differentiate, and give rise to a new tumor (Visvader and Lindeman 2012). Recently, lots of surface markers of CSCs have been identified, including CD133, CD13, CD24, ALDH1A1, CD44, and so on (Henderson *et al.* 2018; Li *et al.* 2018; Marotta *et al.* 2011; Organista-Nava *et al.* 2014; Yang *et al.* 2014). It is still a general strategy to isolate CSCs by FACS and to examine their biological features. There are usually more than one surface marker of CSCs that have been found in a certain tumor, indicating the heterogeneity within CSCs (Haraguchi *et al.* 2010; Yang *et al.* 2008b). The heterogeneity of liver CSCs was proved by single-cell sequencing (Zheng *et al.* 2018). Combination of different markers may be a better strategy for CSC enrichment. Fan lab revealed that CD133^+^CD13^+^ liver CSCs have much stronger self-renewal and tumorigenesis capacities than CD133^+^ CSCs or CD13^+^ CSCs alone (Wang *et al.* 2015).

CSCs, also termed as tumor initiating cells (TICs), are the predominant cells for tumor initiation (O’Brien *et al.* 2007). Accordingly, the tumor initiation assay is a standard and well-accepted method to examine the self-renewal of CSCs (Hermann *et al.* 2007). Gradient numbers of cells are used for tumor observations, and the ratios of CSCs are calculated by extreme limiting dilution analysis (Zhu *et al.* 2015a, b). The sphere formation is another widely used method for CSC detection (Cao *et al.* 2011). In FBS-free medium and ultra-low adherent plates, normal tumor cells die of anoikis, while CSCs can escape from anoikis and propagate into oncospheres. CSCs from many tumor types can generate oncospheres, including breast cancer, liver cancer, colorectal cancer, bladder cancer, and so on (Cao *et al.* 2011; Ponti *et al.* 2005; Ricci-Vitiani *et al.* 2007; Yang *et al.* 2017). The side population is another assay for CSCs. As we know, CSCs play a critical role in drug resistance (Dean *et al.* 2005). Highly expressed many drug-pump molecules such as ABCG2 and CSCs can pump intracellular drugs out of cells to escape drug-induced cytotoxicity. During withdrawal of drugs, survived CSCs can propagate and differentiate into a new tumor, which is termed “tumor relapse” (Merlos-Suarez *et al.* 2011). Taking advantage of this characteristic, researchers developed the side population assay to detect CSCs (Chiba *et al.* 2006).

Recently, several new strategies for CSC study have been established. Genetic lineage tracing is an important tool to examine the self-renewal of CSCs *in vivo* (Meacham and Morrison 2013). Lgr5 and OLFM4 tracing proved that colorectal cancer originated from Lgr5^+^ intestinal stem cells (Barker *et al.* 2009, 2007; Schepers *et al.* 2012; Van der Flier *et al.* 2009). As we know, almost all surface markers of CSCs are also markers of normal tissue stem cells, and CSC-targeted intervention probably blocks tissue hemostasis and renewal. Therefore, it is an urgent issue to identify CSC-specific markers. Two-dimensional mass spectrometry and single-cell RNA sequencing are ideal methods to identify novel markers of CSCs (Zheng *et al.* 2018). It also made sense to deliver CSC-targeting reagents with nanoparticles, which are majorly distributed in tumors due to enhanced permeability and retention (EPR) effects (Gao *et al.* 2004; Schroeder *et al.* 2012; Sun *et al.* 2014). By delivering gene or drugs, some smart and environment-response nanoparticles also emerge as good carriers in CSC targeting (Schroeder *et al.* 2012; Sun *et al.* 2014).

## Signaling pathways for CSCs

The self-renewal of CSCs is maintained under precise regulation, and there are several major signaling pathways in the CSC regulation, including Wnt/β-catenin, Notch, and Hedgehog signaling pathways. The Wnt/β-catenin signaling plays a critical role in many physiological and pathological processes, including development, organ formation, and tumorigenesis (Clevers 2006). As the most important signaling in the regulation of the self-renewal of CSCs, Wnt/β-catenin signaling is activated by β-catenin and TCF, leading to expression of target genes, containing c-MYC, CCND1/2, Axin2, SOX4, TCF7, ASCL2, LGR5, and so on (MacDonald *et al.* 2009). When the Wnt signaling is OFF, β-catenin is located in the cytoplasm and form the APC-degrading complex (Wu *et al.* 2003). When the Wnt signaling is ON, the APC-degrading complex is disrupted and β-catenin translocates into the nucleus, where it associates with TCF/LEF to form the β-catenin-activating complex (Korinek *et al.* 1997). Many inhibitors of the Wnt/β-catenin pathway have been used for intervention of CSCs.

The Notch signaling, another critical modulator for development, also regulates CSC self-renewal (Kopan and Ilagan 2009). When engaged with Notch ligands (DLL1-4), Notch receptors are cleaved by γ-secretase into a stable intracellular domain (NICD), which can translocate into the nucleus and activate the transcription of Notch target genes, including HES family genes, HEY family genes, NRARP, and so on (Mumm and Kopan 2000). Of note, the roles of Notch signaling in CSC self-renewal are controversial, depending on cancer types and Notch receptors. Fan lab showed that in liver CSCs, NOTCH2 is a predominant NOTCH receptor. NOTCH2 is highly expressed in liver CSCs and plays a critical role in the self-renewal maintenance of liver CSCs (Zhu *et al.* 2015b).

The Hedgehog signaling drives progress of basal cell carcinoma, bladder cancer, and other tumors (Li *et al.* 2016; Takebe *et al.* 2011). The activation of Hedgehog signaling is controlled by two receptors, Patched and Smo. The Patched receptor inhibits the activation of Hedgehog pathway and the Smo receptor plays an opposite role. Once engaged with ligands (shh, ihh, dhh), the inhibition of Patched is relieved and Smo is activated, and Hedgehog target genes are consequently expressed (Katoh and Katoh 2006). Similar with Wnt/β-catenin and Notch signaling pathways, the Hedgehog activation is also well regulated. For instance, GALNT1, a glycotransferase highly expressed in BCMab1^+^CD44^+^ bladder CSCs, can activate Hedgehog signaling through O-linked glycosylation of SHH and promote the self-renewal of bladder CSCs (Li *et al.* 2016).

Besides Wnt/β-catenin, Notch, and Hedgehog signaling pathways, other signaling pathways are also involved in the self-renewal of certain tumors. For example, lung CSCs secrete CSF and c-Kit to drive their self-renewal through an autocrine manner (Levina *et al.* 2010). PTEN pathway plays a critical role in the self-renewal regulation of esophageal CSCs and breast CSCs (Li *et al.* 2011; Zhou *et al.* 2007). Yap1 and Rspo are predominant modulators for colorectal CSCs (Barry *et al.* 2013). In addition, Yap1 signaling modulates the cell-fate and plasticity of liver CSCs (Zhu *et al.* 2016b).

## Genetic and epigenetic regulation of CSCs

Accumulating evidence shows that many genetic and epigenetic factors are involved in the regulation CSC self-renewal. As we know, transcription factors (TFs) are critical modulators in cell-fate determination. Oct4, c-Myc, Nanog, and Klf4 overexpression can convert fibroblast cells into induced pluripotent stem (iPS) cells (Takahashi and Yamanaka 2006). Similar to iPS cells, CSCs can also self-renew and differentiate. Oct4, c-Myc, Nanog, and Klf4 were also identified as critical regulators in the maintenance of CSC self-renewal (Lee *et al.* 2011; Tseng *et al.* 2014; Zhu *et al.* 2015a). Some TFs involved in development are also required for the maintenance of CSCs, including Zic2, Notch2, and so on (Zhu *et al.* 2015a, b).

In fact, tumorigenesis is a process of oncogenic reprogramming, and many chromatin remodeling complexes are dysregulated in cancer cells and CSCs (Wang *et al.* 2007). As a driver factor in tumorigenesis, the chromatin remodeling becomes a critical target for cancer and CSC elimination (Jones and Baylin 2007). It has been reported that the SWI/SNF complex is involved in oncogenic reprogramming and CSC self-renewal (Klochendler-Yeivin *et al.* 2002). The SWI/SNF complex can be formed into BRG1-contained SWI/SNF complex and BRM-contained SWI/SNF complex. The BRG1-contained SWI/SNF complex is increased in liver tumorigenesis, whereas the BRM-contained SWI/SNF complex is decreased. This switch between BRG1- and BRM-contained SWI/SNF complex plays a critical role in liver tumorigenesis and liver CSC self-renewal (Zhu *et al.* 2016b). The mutation of ARID1A, a component of SWI/SNF complex, also plays an important role in liver tumorigenesis and bladder tumorigenesis, and drives the self-renewal of liver CSCs and bladder CSCs as well (Fujimoto *et al.* 2012; Yang *et al.* 2017).

Many components of PRC2 complex are frequently mutated in various tumors. EZH2 is the core component of PRC2 and highly expressed in many solid tumors (Takawa *et al.* 2011). EZH2 depletion results in decreased breast CSCs and liver CSCs (Kleer *et al.* 2003; Zhu *et al.* 2016a). In contract, EZH2 loss of function mutation also drives tumorigenesis in acute lymphoblastic leukemia (Ntziachristos *et al.* 2014). Of note, EZH2 also promotes CSC self-renewal through a PRC2-independent manner. Xu *et al.* revealed that the oncogenic role of EZH2 in prostatic cancer is PRC2-independent (Xu *et al.* 2012). The non-classic role of EZH2 was validated in glioblastoma CSCs and liver CSCs, in which EZH2 exerts its role through STAT3 and β-catenin methylation (Kim *et al.* 2013; Zhu *et al.* 2016a). PRC1 complex is also involved in tumorigenesis, and the expression of its component BMI1 is related to the prognosis of many kinds of tumors (Laugesen and Helin 2014). BMI1 overexpressing head and neck squamous cell carcinoma showed enhanced metastasis and CSC-like characteristics (Yu *et al.* 2011).

The NURD complex is another critical remodeling complex that participates in the CSC self-renewal (Lai and Wade 2011). HDAC1 and HDAC2, two components of the NURD complex, are highly expressed in tumor patients with bad prognosis (West and Johnstone 2014). In many murine tumor models, HDAC1 and HDAC2 blockade inhibits tumor progress and CSC self-renewal (West and Johnstone 2014). However, the role of NURD complex in tumorigenesis and CSC self-renewal is controversial, depending on different components and tumor types. For example, HDAC1 and HDAC2 are highly expressed in breast cancer and promote tumor progresses, and LSD1 is lowly expressed in breast cancer and inhibits tumor progress (Lai and Wade 2011; Wang *et al.* 2009; West and Johnstone 2014). This inconsistency is also reflected by clinical applications. Although HDAC inhibitors inhibit tumor progress, some inhibitors may promote the propagation of certain tumors (Santoro *et al.* 2013).

Besides the SWI/SNF, PRC, and NURD complexes, some other chromatin remodeling complexes also participate in the tumorigenesis and CSC self-renewal. The NURF complex is highly expressed in liver cancer and liver CSCs, and drives the self-renewal of liver CSCs through OCT4 (Zhu *et al.* 2015a). Single-cell sequencing of bladder CSCs, non-CSCs, and normal bladder cells revealed that MLL2 promotes the self-renewal of bladder CSCs (Yang *et al.* 2017). Histone acetyltransferase MOZ and MORF are critical modulators for the self-renewal of hematopoietic stem cells and leukemia CSCs (Yang and Ullah 2007).

Histone modification is also involved in the tumorigenesis and CSC self-renewal. In acute leukemia CSCs, H3K4me3 and H3K27me3 were enriched in the regions of CSC-associated genes (Yamazaki *et al.* 2013). The H3K4me3 levels in *Oct4*, *Yap1,* and *TCF7* promoters are also related to the self-renewal of liver CSCs (Wang *et al.* 2015; Zhu *et al.* 2016a, b). Some modifications of histone variants are also related to the CSC self-renewal. The acetylation of H2A.Z (acH2A.Z) and methylation are involved in transcriptional repression of prostate cancer (Valdes-Mora *et al.* 2012). In addition to the modifications of specific regions, the total modification levels of H3K18ac and H3K4me2 are also correlated with prostate relapse, and the levels of H3K9ac, H3K18ac, H4K12ac, H4K16ac, H3K4me2, H4K20me3, and H4R3me2 are related to breast tumorigenesis as well (Elsheikh *et al.* 2009; Seligson *et al.* 2005).

Non-coding RNAs are also modulators for the CSC self-renewal, including microRNA, snoRNA (small nucleolar RNA), circRNA (circular RNA), lncRNA (long non-coding RNA), and so on. MicroRNA let-7 (Mir-let-7) is the first identified microRNA involved in CSC self-renewal. Mir-let-7 is lowly expressed in breast CSCs, and inhibits the self-renewal of breast CSCs and breast tumorigenesis through H-Ras and HMGA2 (Yu *et al.* 2007). Mir-200c is another lowly expressed microRNA in breast CSCs, and suppresses breast CSC self-renewal through BMI expression (Shimono *et al.* 2009). Moreover, Mir-200c also participates in cell-fate decision of breast CSCs and non-CSCs (Shimono *et al.* 2009). In addition, Mir-34a inhibits the self-renewal of prostatic CSCs and prostatic cancer metastasis (Liu *et al.* 2011). Mir-181 is a modulator for the self-renewal of liver CSCs (Ji *et al.* 2009).

**Fig. 1:**
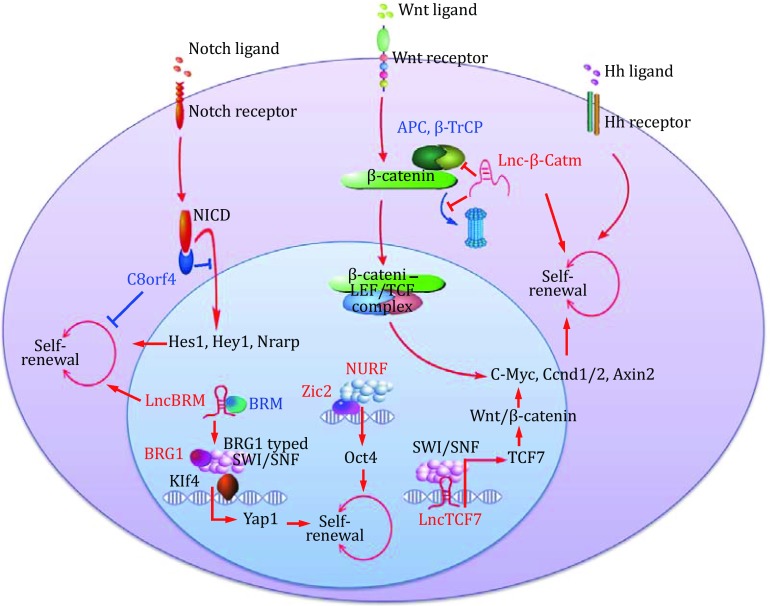
Newly identified modulators of liver CSC self-renewal. LncTCF7, lnc-β-catm, lncBRM, C8orf4, and Zic2 were identified as critical liver CSC regulators by Fan lab (Hermann *et al.* 2007; Takawa *et al.* 2011; Yang *et al.* 2008a, b; Zhu *et al.* 2015a, b, 2016a, b). The newly identified positive regulators are shown in *red* and negative regulators in *blue*

LncRNAs emerge as critical modulators in tumorigenesis and CSC self-renewal. MALAT-1 is highly expressed in pancreatic cancer and pancreatic CSCs, and its expression confers pancreatic cancer cell stem-like characteristics (Jiao *et al.* 2015). PVT1 is copy-number gained in various tumors, accompanied with PVT1 overexpression, which promotes the stability of c-Myc and thus initiates tumorigenesis and CSC self-renewal (Tseng *et al.* 2014). Fan lab identified several lncRNAs in liver CSCs that are involved in their self-renewal maintenance (Fig. 1). LncTCF7 is highly expressed in liver CSCs and required for the self-renewal of liver CSCs. Mechanistically, LncTCF7 recruits the SWI/SNF complex to initiate TCF7 expression and subsequently initiates Wnt/β-catenin activation (Wang *et al.* 2015). Lnc-β-catm promotes the interaction between β-catenin and EZH2, which further methylates β-catenin and promotes its stability, leading to Wnt/β-catenin signaling activation in liver CSCs (Zhu *et al.* 2016a). LncBRM binds specifically to BRM, but not BRG1, promoting the assembly of BRG1-contained SWI/SNF complex. The BRG1-contained SWI/SNF complex further initiates Yap1 signaling and finally sustains the liver CSC self-renewal (Zhu *et al.* 2016b). Guarnerio *et al.* found many fused circular RNAs in leukemia drive leukemia tumorigenesis together with oncogenes (Guarnerio *et al.* 2016). Finally, sno-lncRNA SLERT can promote the transcription activity of ribosome RNA and initiates tumorigenesis (Xing *et al.* 2017).

Many genetic and epigenetic modulators have been identified in the regulation of CSC self-renewal; however, the precise regulatory mechanisms of CSC are still elusive. Some genome-scaled unbiased screening technologies, including shRNA screening, CRISPR screening, and CRISPRi screening, have been widely used to identify functional genes (Liu *et al.* 2017; Shalem *et al.* 2014). To our knowledge, similar screenings have not been used for CSC research, and we believe that many potential modulators will be identified in the near future by using these functional screening techniques.

## CSC niches

CSCs reside in their niches, which sustain the self-renewal of CSCs and triggers tumorigenesis. The CSC niche contains niche cells and cytokines, and can be divided into inflammatory niche, perivascular niche, premetastatic niche, extracellular matrix, and neighboring cells.

In the inflammatory niche, tumor-associated macrophages and CD4^+^ T cells secrete TNFα and activate NF-κB signaling of CSCs to induce expression of Slug, Snail, and Twist, and consequently drive epithelial-mesenchymal transition (EMT) and CSC self-renewal (Liu *et al.* 2017).

The location of CSCs in tumor bulk has been an important issue for a long time. The CSCs were first observed to be located near from blood vessels. Head CSCs were found to be in contact with vascular endothelial cells directly, and the number of CSCs is related to vascular intensity (Calabrese *et al.* 2007). When co-cultured with vascular endothelial cells, CSCs form oncospheres 5-fold as large as CSCs alone (Calabrese *et al.* 2007). Neurospoagioma CSCs also reside near from blood vessels, where are supported by vessel-derived Hedgehog, Notch, and PI3K molecules (Charles *et al.* 2010). The perivascular niche protects CSCs from radiation-induced damage, and initiates the self-renewal of CSCs (Charles *et al.* 2010). Meanwhile, vascular endothelial cells sustain the self-renewal of CSCs through the VEGF-Nrp1 signaling pathway (Beck *et al.* 2011).

Hypoxia is a typical characteristic of tumors and also serves as CSC niches. Hypoxia drives the self-renewal of CSCs through ROS and TGFβ signaling, and also protects CSCs from drug-induced and radiation-induced cell death (Scheel *et al.* 2011). Hypoxia-induced factor 1α (HIF1α) directly activates Notch signaling and thus drives CSC self-renewal in many solid tumors (Quail *et al.* 2012). HIF1α also keeps CSCs in a quiescent state, reduces DNA damage, and finally maintains the self-renewal of CSCs.

CSCs in primary locus and metastasis locus share similar transcription landscapes. There are also large similarity between CSCs and circulating tumor cells, which are critical for tumor metastasis. In addition, circulating tumor cells highly expressing CSC markers were also identified recently. These observations proved the relationship between CSCs and tumor metastasis (Yachida *et al.* 2010). The premetastatic niche contains six characteristics, including immune repression, inflammation, angiogenesis, lymphangiogenesis, organotropism, and reprogramming, which drives tumor metastasis and CSC self-renewal (Liu and Cao 2016). Moreover, the premetastatic niche also contains abundant vessels, niche cells, and factors, which support the survival and plasticity of CSCs (Takebe *et al.* 2011). In the lung metastasis of breast cancer, CSCs can induce periostin expression in lung fibroblast, which further drives the self-renewal of CSCs through engaging with Wnt ligands (Kitamura *et al.* 2015).

**Fig. 2:**
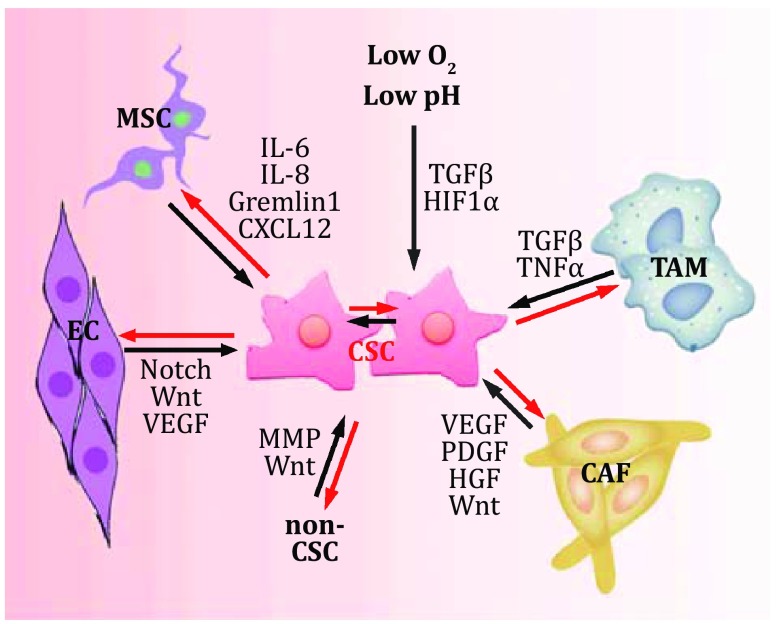
Mutual interactions between CSCs and CSC niches. Main niche cells and factors for CSC self-renewal are shown. CSC: cancer stem cell, TAM: tumor-associated macrophage, CAF: cancer-associated fibroblast, MSC: mesenchymal stem cells, EC: endothelial cells

There are various kinds of cells near from CSCs, which support CSC self-renewal with nutrition and cytokines (Fig. 2). Mesenchymal stem cells (MSCs), cancer-associated fibroblasts (CAFs), tumor-associated macrophages (TAMs), and non-stem cancer cells play critical roles in the maintenance of CSC self-renewal. MSCs are multiple functional cells and can secrete many cytokines to promote the CSC self-renewal. MSCs can activate NF-κB signaling and drive CSC self-renewal by secreting CSCL12, IL-6, and IL-8 as well. Meanwhile, MSCs also secrete BMP antagonists to sustain CSCs in an undifferentiated state (Davis *et al.* 2015). In breast cancer, MSCs induce Mir-199a expression in cancer cells, which further inhibits FoxP2 expression and drives CSC self-renewal (Cuiffo *et al.* 2014). In tumors, endothelial cells and CSCs can convert fibroblasts to CAFs. CAFs secrete extracellular matrix, including VEGF, PDGF, HGF, and CXCL12, which drive the activation of Wnt and Notch signaling pathways for the self-renewal maintenance of CSCs (Kalluri and Zeisberg 2006). CAFs also remodel extracellular matrix, promote EMT, and drive CSC self-renewal through secreting metalloproteases such as MMP2, MMP3, and MMP9. Recently, Song lab identified a new subgroup of CAFs termed CD10^+^GPR77^+^ CAFs, which secrete IL-6 and IL-8 to activate NF-κB signaling in CSCs, and consequently promote the propagation of breast cancer and lung cancer, drug resistance, and CSC self-renewal (Su *et al.* 2018). In addition, CSCs recruit macrophages, medullary precursor cells and MSCs to form a paracrine niche, which maintains the self-renewal of CSCs as well.

## Targeting strategies against CSCs and future challenges

CSCs are considered as the origin of tumorigenesis, metastasis, drug resistance, and relapse. CSCs are in quiescent state and survive in response to many drugs that target tumor propagation (Kurtova *et al.* 2015). Meanwhile, CSCs can pump drugs out of cells owing to high-expression drug-pump molecules. Especially, CSCs can escape from drug-induced cell death. Unlike many tumor cells, CSCs efficiently escape from anoikis, which is prerequisite for tumor metastasis (Kreso and Dick 2014). During withdrawal of drugs, survived CSCs can propagate and differentiate into a new tumor. Therefore, targeting CSCs is a big challenge for tumor elimination.

As mentioned above, many surface markers of CSCs have been identified, and these markers become first choices for CSC targeting. CD13 antibody targeting liver CSCs can efficiently eliminate liver CSCs (Haraguchi *et al.* 2010). BCMab1 antibody targeting bladder CSC surface marker Integrin α3β1 can inhibit the self-renewal of bladder CSCs and effectively suppress bladder cancer propagation (Li *et al.* 2014). Some important membrane proteins also serve as targets for CSC elimination. Rspo3 is highly expressed in colorectal cancer and plays a critical role in the self-renewal of intestinal stem cells and colorectal CSCs, and its blockade antibody has effective intervention on colorectal CSCs and colorectal tumors (Storm *et al.* 2016). The Wnt/β-catenin, Hedgehog, Notch, BMP, and Pten pathways are also used as targets for CSC elimination, and the inhibitors of these signaling pathways can repress the CSC self-renewal. Although CSCs resist to traditional radiotherapy and chemotherapy, they are sensitive to certain therapies. For example, rapamycin, an inhibitor of mTOR signaling pathway, can eliminate CSCs of PTEN-deficient leukemia (Yilmaz *et al.* 2006). G-CSF treatment on ALL CSCs can promote entrance of cell cycle, and thus increase the sensitivity of ALL CSCs to chemotherapy (Kreso and Dick 2014). Finally, BMP4 can convert glioma CSCs into normal glia cells (Gargiulo *et al.* 2013).

Targeting CSC niches emerges as a new therapy for CSC clearance. HIF1α and HIF2α are good targets for glioma and glioma CSCs (Soeda *et al.* 2009). VEGF targeting antibody Bevacizumab suppresses the self-renewal of CSCs and effectively inhibits tumor propagation and metastasis (Ye *et al.* 2014). Inhibition of CCR2 and M-CSF in pancreatic cancer decreases the numbers of macrophages and CSCs (Mitchem *et al.* 2013). The antibody against fibronectin receptor Integrin α4β1 inhibits the protection of CSCs resisting to chemotherapy (Kaplan *et al.* 2005). Moreover, CSCs can also serve as a target for immunotherapy, a promising therapy for tumor treatment (Codd *et al.* 2018).

However, many CSC markers are also the markers of normal tissue stem cells and progenitor cells. For instance, LGR5, a marker of colorectal CSC, is also a marker of intestinal stem cells (Barker *et al.* 2007). CD133 and CD44 are CSC markers in many tumors, and they are also markers of many tissue progenitor cells. Wnt/β-catenin, Notch, and Hedgehog pathways not only participate in the self-renewal of CSCs, but also play critical roles in the maintenance modulation of normal tissue stem cells (Yang *et al.* 2008a). With the development of modern methodology, some specific markers for CSCs must be identified and can be used for CSC targeting in the future.

The heterogeneity of CSCs themselves is another challenge for CSC targeting. CSCs also contain various subgroups of cells according to singe cell sequencing and experimental data. Fan lab revealed a multiple origin of bladder CSCs through single-cell sequencing (Yang *et al.* 2017). Several markers of CSCs have been identified for a certain tumor type (Wang *et al.* 2015). CSC heterogeneity surely increases the difficulty for CSC targeting. Identifying all CSC subsets using single-cell RNA sequencing is needed for the study of CSC biology. In addition, the plasticity of CSCs is another layer difficulty for CSC targeting. CSCs have plastic capacity, and non-CSCs can transdifferentiate into CSCs once the primary CSCs are eliminated. Moreover, the newly formed CSCs can be also resistant to CSC-targeted therapies. As maintained above, targeting tumor cells with chemotherapy leads to the emerging of drug-resistant tumor cells derived from CSCs, and similarly, targeting CSCs can induce therapy-resistant CSCs due to non-CSC dedifferentiation. Therefore, the switch between CSCs and non-CSCs implies the necessity of combination of CSC-targeted therapy with traditional therapy together.
